# P-1960. Desirability of Outcome Ranking (DOOR) Analysis of Isavuconazole Relative to Voriconazole for the Treatment of Invasive Mold Disease (IMD)

**DOI:** 10.1093/ofid/ofaf695.2127

**Published:** 2026-01-11

**Authors:** Thomas L Holland, David R Walker, Jun Liu, Yana Labinov, Thomas Lodise

**Affiliations:** Duke University, Durham, NC; Astellas Pharma Global Development Inc., Northbrook, Illinois; Astellas Pharma Global Development Inc., Northbrook, Illinois; Astellas Pharma Global Development Inc., Northbrook, Illinois; Albany College of Pharmacy and Health Sciences, Stratton, VA, United States, Stratton, VA

## Abstract

**Background:**

In SECURE, a randomized, Phase 3, double-blind trial, isavuconazole was non-inferior to voriconazole for primary treatment of suspected IMD. To better understand the association between treatment benefits and harms, a within-patient analysis using DOOR was performed.

**Figure:**
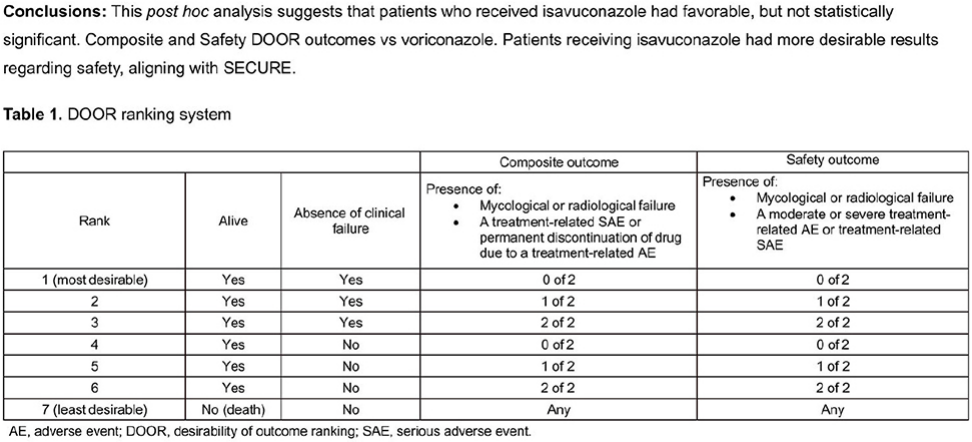

**Figure:**
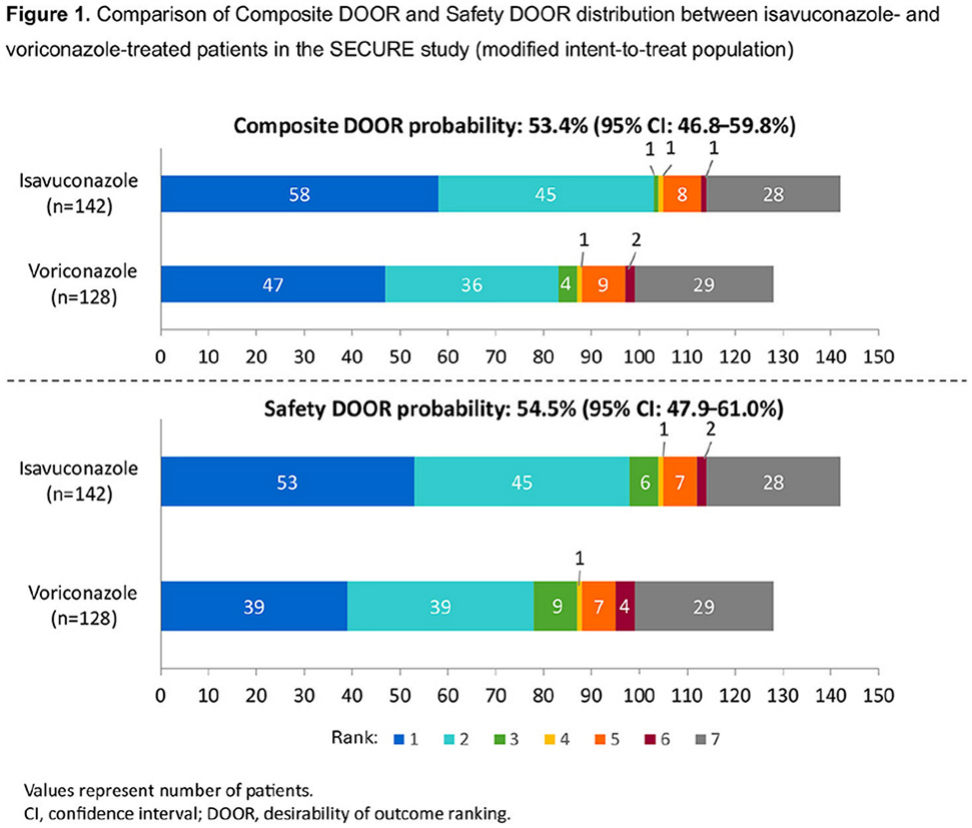

**Methods:**

Patients in SECURE with proven or probable IMD as determined by the data review committee were analyzed. Patients were assigned a mutually exclusive DOOR rank, from 1 (most desirable) to 7 (least desirable) (Table 1). Two DOOR ranking systems served as primary endpoints: Composite DOOR and Safety DOOR. The DOOR distribution for each outcome was compared between treatment groups using the Wilcoxon Mann–Whitney *U* statistic; DOOR probability >50% indicates a more favorable outcome with isavuconazole vs voriconazole. DOOR distributions were also compared between treatments in pre-specified subgroups of age and sex. The probability for each DOOR component (clinical failure; mycological or radiological failure; treatment-related serious adverse event [TRSAE]; discontinuation of treatment due to a treatment-related adverse event [TRAE]; moderate/severe TRSAE/TRAE; and death) was compared between treatments. A DOOR partial credit analysis was performed to compare mean partial credit scores between treatments for each outcome.

**Figure:**
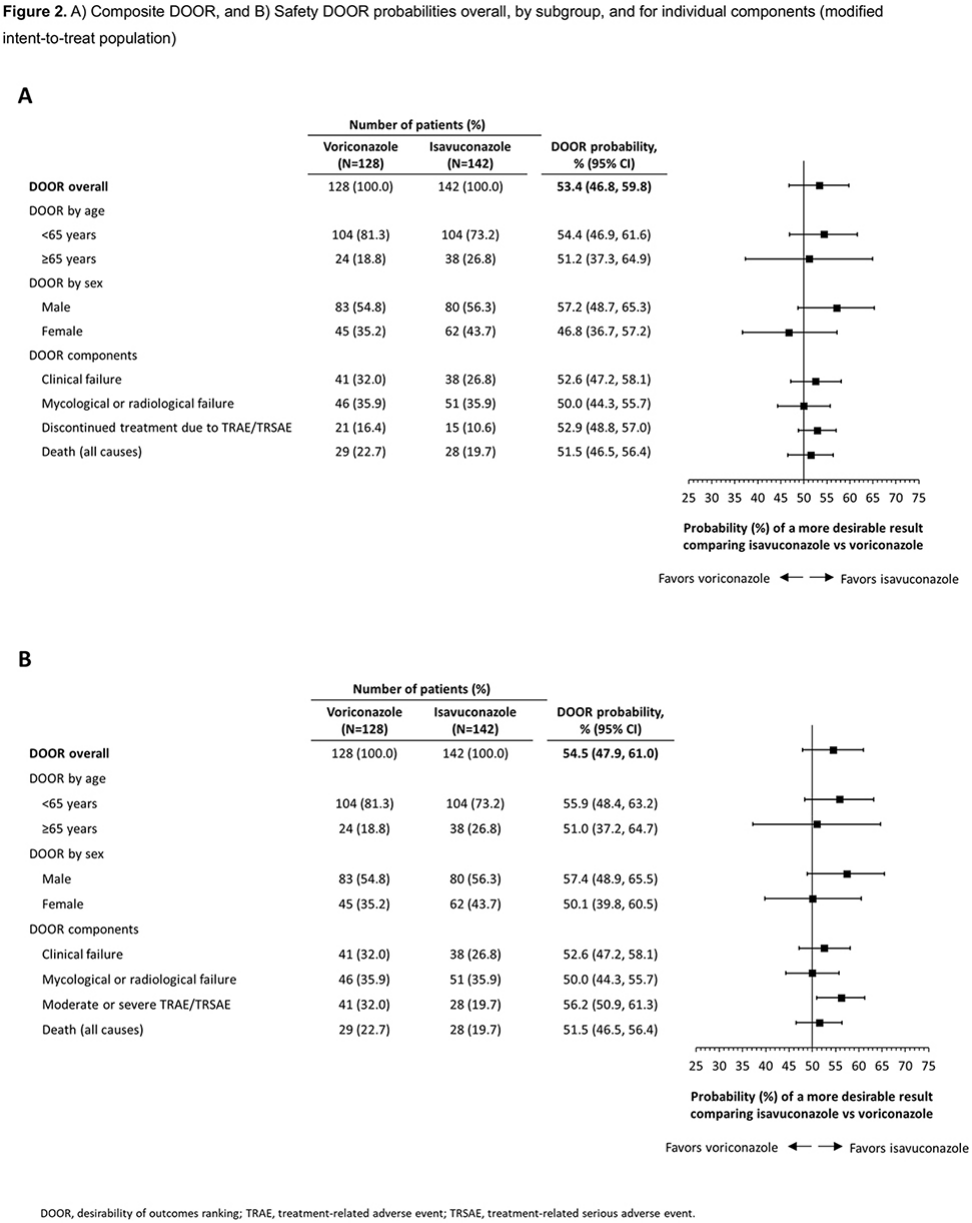

**Figure:**
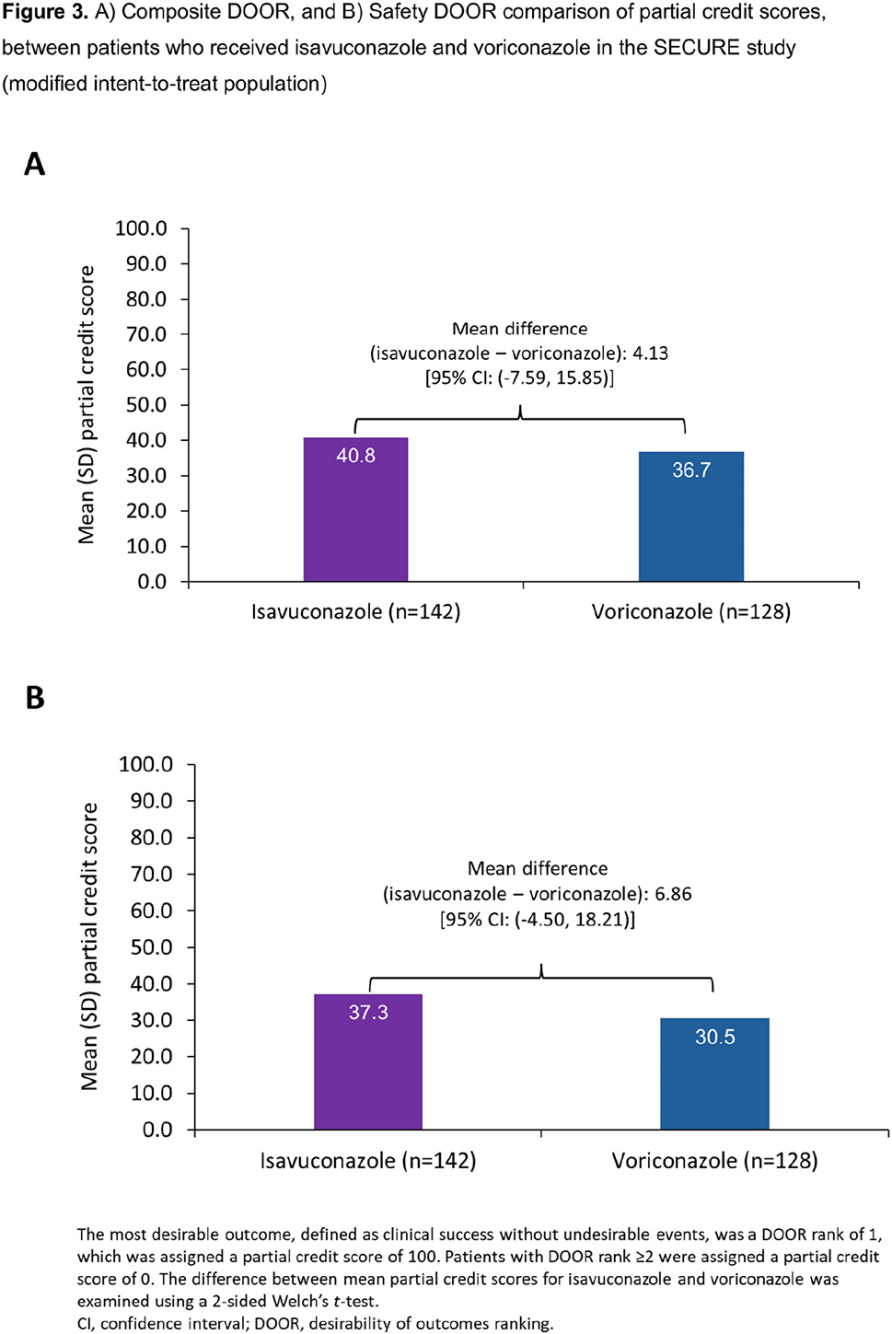

**Results:**

142 isavuconazole- and 128 voriconazole-treated patients were included. The probability of a more favorable Composite DOOR outcome with isavuconazole vs voriconazole was 53.4% (95% confidence interval [CI]: 46.8–59.8%) (Figs. 1 and 2A). In the Safety DOOR analysis, the probability was 54.5% (95% CI: 47.9–61.0%) (Figs. 1 and 2B). In the Safety DOOR, there was a 56.2% probability of achieving a more favorable outcome with isavuconazole vs voriconazole for the Safety component of moderate/severe TRAE/TRSAE (95% CI: 50.9–61.3%). Partial credit scores favored isavuconazole in both DOOR analyses, but did not reach statistical significance (Fig. 3).

**Conclusion:**

This *post hoc* analysis suggests that patients who received isavuconazole had favorable Composite and Safety DOOR outcomes vs voriconazole, although differences were not statistically significant. Patients receiving isavuconazole had more desirable results regarding safety, aligning with findings from SECURE.

**Disclosures:**

Thomas L. Holland, MD, MSc-GH, FIDSA, Affinivax: Advisor/Consultant|Aridis: Advisor/Consultant|Basilea Pharmaceutica: Advisor/Consultant|Concert: Advisor/Consultant|Lysovant: Advisor/Consultant|NIH Antibacterial Resistance Leadership Group and the Karius Adjudication Committee: Grant/Research Support|Pfizer: Advisor/Consultant|PSI: Advisor/Consultant|Spero Therapeutics: Data Safety Monitoring Board|Staphylococcus aureus bacteraemia Network Adaptive Platform (SNAP trial): Data Safety Monitoring Board|UpToDate: Royalties David R. Walker, PhD, Astellas: Former employee Jun Liu, PhD, Astellas: Current employee Yana Labinov, PharmD, Astellas: Current employee Thomas Lodise, Jr., PharmD, PhD, GSK: Advisor/Consultant

